# A simple immunohistochemical bio-profile incorporating Bcl2 curbs those cases of invasive breast carcinoma for which an Oncotype Dx characterization is needed

**DOI:** 10.1371/journal.pone.0217937

**Published:** 2019-06-03

**Authors:** Claudio Ceccarelli, Antonio De Leo, Pasquale Chieco, Claudio Zamagni, Alice Zamagni, Daniela Rubino, Mario Taffurelli, Donatella Santini

**Affiliations:** 1 Department of Experimental, Diagnostic and Specialty Medicine–DIMES, St.Orsola-Malpighi Hospital, University of Bologna, Bologna, Italy; 2 Unified Centre for Biomedical Applied Research–CRBA, St.Orsola-Malpighi Hospital, University of Bologna, Bologna, Italy; 3 Breast Medical Oncology Unit, Department of Hematology and Oncology, St.Orsola-Malpighi Hospital, Bologna, Italy; 4 Radiation Oncology Unit, S.Orsola-Malpighi Hospital, Bologna, Italy; 5 Department of Medical and Surgical Sciences–DIMEC, St.Orsola-Malpighi Hospital, University of Bologna, Bologna, Italy; 6 Surgical Pathology Unit, Department of Diagnostic and Prevention Medicine, St.Orsola-Malpighi Hospital, Bologna, Italy; University of Toronto, CANADA

## Abstract

**Aim:**

Our goal has been to evaluate the importance that the incorporation of Bcl2 in the ER/PGR/Her2/Ki67 bio-profile can have as predictor of the Oncotype Dx categories.

**Material and methods:**

156 consecutive cases of HR+/Her2- pN0/1 primary breast carcinoma were sent to the Oncotype Dx test. Immunohistochemical determination of Bcl2/ER/PGR/Ki67/Her2 expression was evaluated for each case. After the selection of the appropriate cut-off values for PGR and Ki67, explorative as well as confirmative statistical analyses were performed to build and validate predictive risk-of-recurrence immunohistochemical only bio-profiles.

**Results:**

The predictive capacity of these immunohistochemical profiles was compared with both traditional and TAILORx Oncotype Dx risk class classification. This comparison showed that immunohistochemical bio-profiles select those cases not associated with high risk-of-recurrence of disease (luminal-A/B and luminal A/B Bcl2) and those that are instead at high risk and therefore worthy of chemotherapy (luminal-B ki67 and luminal-B Bcl2/Ki67), strongly suggesting to only submit PGR-positive/Bcl2-Ki67 altered cases to Oncotype Dx, thus reducing the number of cases to be tested.

**Conclusions:**

Our results indicate that the addition of Bcl2 to an immunohistochemical bio-profile definitely improves its predictive capacity to correctly select which cases to send to the Oncotype Dx test. We have also suggested that institutions with a significant number of breast carcinomas sent to the Oncotype Dx test can use these latter to derive their own PGR and Ki67 cut-off values, overcoming the drawbacks of sharing common inter-laboratory values. Validation of these bio-profiles as predictors of the Oncotype Dx categories is ongoing in a prospective series of new cases.

## Introduction

The most recent TNM AJCC classification (8th edition) [1] has defined breast cancer as a group of diseases with different molecular characteristics, and promoted the addition of specific biomarkers to guide personalized systemic therapies. In the light of this perspective, the determination of ER, PGR and Her2 expression using immunohistochemistry (IHC) or multigene panels is today part of TNM AJCC prognostic staging. Risk assessment is crucial to plan correct therapies in breast cancer, and in the light of this need, it is important to properly distinguish those Hormonal Receptor (HR)-positive/Her2-negative cases that required chemotherapy. This risk assessment has gained significant improvement by the introduction of Oncotype Dx molecular assay (Genomic Health, Redwood City, CA, USA). This molecular 21-gene assay quantifies the risk of distant recurrence at 10 years from diagnosis (Recurrence Score—RS) giving a retrospective (NSABP B14 and B-20) and prospective (TAILORx) validated indication of the potential benefit of chemotherapy for those HR-positive/Her2-negative N0 cases [2,3]. In addition, the Oncotype Dx report contains qRT-PCR results for ER, PGR and Her2, all of which are further classified using specific cut-off values.

Similarly, in routine practice, the integration of ER, PGR and Her2 values determined with IHC and quantified according to specific cut-off values support luminal classification. The recommendations of the ASCO/CAP guideline for IHC hormone testing stated that 1% of immunostained cells are the correct cut-off value to distinguish between positive and negative ER and/or PGR cases [4]. However, as far as the evaluation of PGR is concerned, this statement is not completely shared by oncologists; indeed a 20% cut-off was proposed at the St. Gallen consensus meeting in 2013, and a 10% cut-off is considered valid by several authors [5–8]. With regard to Her2 IHC classification, the ASCO/CAP recommendations allow us to distinguish negative (score 0/1+) from equivocal (score 2+) or positive (score 3+) cases; equivocal scores will then be changed to negative or positive using *in situ* hybridization. Further, Ki67 IHC could also be of help to distinguish luminal-A from luminal-B cases [9]. The relevance of Ki67 is supported by its proven relationship with the Oncotype Dx risk-of-recurrence assessment [10–13]. However, its prognostic/predictive role is hindered by at least two important drawbacks: reproducibility and selection of the cut-off value [9,14–17].

In our institution, Bcl2 was added to the above-mentioned IHC prognostic markers to better distinguish between luminal A/B subtypes. Bcl2 presence was positively associated with favorable prognostic features in breast cancer, emerging as an independent predictor for either DFS or OS [18–21]. It is noteworthy that Bcl2 is also part of the 21-gene Oncotype Dx signature.

Being part of the PONDx Survey, we sent to the Oncotype Dx test all the early HR-positive/Her2-negative, pT1/2, pN0/1 luminal cases diagnosed in 2017. We then decided to compare our established IHC bio-profile with the Oncotype Dx Recurrence Score. The purpose of the present study is to evaluate whether a proper IHC profile could be used as a reliable screening tool to predict Oncotype Dx risk categories, helping in identifying those patients who really need this test for treatment decision in early breast cancer.

## Materials and methods

The study was approved by the CE-AVEC (Comitato Etico—Area Vasta Emilia Centro) register n° 668/2018/Oss/AOUBo. All patients signed an informed consent permitting the use of the data necessary for the study.

### Patients characteristics

In our institution, 156 consecutively patients diagnosed with HR-positive/Her2-negative, pN0/1, pT1/2 early breast carcinoma were referred to Oncotype Dx testing by the Breast Cancer Multidisciplinary Team from 12^th^ December 2016 to 22^th^ December 2017. The immunohistochemical bio-profile was integrated into the diagnosis. Patients mean age was 61.8 years (range 35–93). The majority of cases presented a diagnosis of invasive carcinoma No Special Type (126 cases– 80.8%), and pT1c (47.7%) pN0 (70.1%) pathological stage. Patients characteristics were reported in Table 1. The tumors were histologically classified according to WHO 2012 criteria. Tumor staging (pTN) was defined following TNM AJCC classification [1]. Tumor grading was performed according to Elston & Ellis criteria. Axillary lymph node status was evaluated following the sentinel node approach.

**Table 1:** Patients and tumor characteristics.

Age (years)	Mean ± S.D. 61.8±12.9	Range 35–93	Cases ≤ 50 years 38	Cases > 50 years 118
**Histologic subtype**	**IC-NST** 126 (80.8%)	**ILC** 25 (16.0%)	**ICDL** 5 (3.2%)	
**Tumor Grade**	**G1** 67 (43.2%)	**G2** 70 (45.2%)	**G3** 18 (11.6)	
**Tumor size (mm)**	**Mean** **±** **S.D.** 15.6±8.1	**Median** 13.5		
**pTN stage**	**T1a** 3 (1.9%)	**T1b** 52 (33.5%)	**T1c** 74 (47.7%)	**T2** 26 (16.9%)
	**N0** 108 (70.1%)	**Nmic** 14 (9.1%)	**N1** 32 (20.8%)	

IC-NST = Invasive Carcinoma No Special Type; ILC = Invasive Lobular Carcinoma; ICDL = Invasive Carcinoma with mixed Ductal and Lobular features

### Immunohistochemistry

Following surgical resection, tissues were sent to the Surgical Unit for histopathologic examination by a dedicated pathologist. Formalin-fixed (12-72h), paraffin-embedded tumor sections were obtained and processed in a Benchmark Ultra immunostainer (Ventana Medical Systems, USA) for ER, PGR, Bcl2, Ki67, and Her2 determination (S1 Table).

Visualization of the immunological reaction was obtained with OptiView DAB Detection kit for ER, PGR, Bcl2 and ki67 staining or with UltraView DAB Detection kit for Her2 staining; slides were counterstained with haematoxylin and bluing reagents.

The percentage of ER, PGR, and Ki67 stained cells was quantified using image cytometry with the IMAGE Pro Plus 5.1 software (Media Cybernetics Inc., USA). For ER and PGR determination the entire section was quickly examined at 10x and then at least twenty-five 200x representative fields scattered in the section were selected, captured, and examined with the software. Ki67 was evaluated on the entire invasive front, counting at least 5.000 cells per case. A labelling index (%Li) was obtained for each of these parameters and expressed as percentage of positive cells on total neoplastic counted cells. Bcl2 immunohistochemical expression was quantified modifying a previously validated semiquantitative method based on its distribution and intensity in neoplastic cells [18]. Cases were classified as: Bcl2 High = presence of a moderate to intense homogeneous staining in all cancer cells; Bcl2 Low = all the remaining cases.

### Oncotype Dx

Sections for Oncotype Dx testing were obtained from the same formalin-fixed, paraffin-embedded tissue block sectioned for immunohistochemical bio-profile. Cases were referred to Oncotype Dx assay after a multidisciplinary meeting evaluation, considering clinical, pathological, and bio-pathological conventional prognostic parameters. The RS results were classified into Low- (L), Intermediate-(I), and High-risk (H) group according to both traditional (RS<18; RS 18–30; RS>30) and the recently proposed TAILORx trial (RS<11; RS 11–25; RS>25) cut-off values [3]. Oncotype (-Dx) ER, PGR, and Her2 qRT-PCR values and their relative classification were also recorded.

### Statistical analysis

The aim of statistical analysis was to verify the extent to which a panel of immunohistochemical markers correlates with the classes of risk obtained with Oncotype DX. Statistical analysis includes Pearson’s Correlation, Concordance and Multinomial logistic regression. Principal component analysis (PCA) for continuous variables and multiple correspondence analysis (MCA) for categorical variables were included for exploratory purposes. PCA analytical techniques are useful to explore association among variables while MCA explore association among different categories [22]. Multinomial Logistic regression using Jackknife resampling procedure [23] was conducted with Stata software v 11.0 (Statcorp, USA).

## Results

### Oncotype Dx cases classification

The Oncotype Dx RS values of our 156 cases were classified according to both traditional as well as TAILORx cut-off values. Using the traditional classification, 106 (67.9%) cases were at Low risk, 42 (26.9%) cases were at Intermediate risk, and 8 (5.2%) cases were at High risk. Using the recently validated TAILORx cut-off values cases were classified as: 40 (25.6%) at Low risk, 102 (65.4%) at Intermediate risk, and 14 (9.0%) at High risk, showing the expected slippage of many Low-risk cases towards the Intermediate risk due to the reduction of cut-off values introduced by the TAILORx trial.

### Correlations between immunohistochemical markers and Oncotype Dx values

#### ER, PGR, and Her2 evaluation

**Fig 1:**
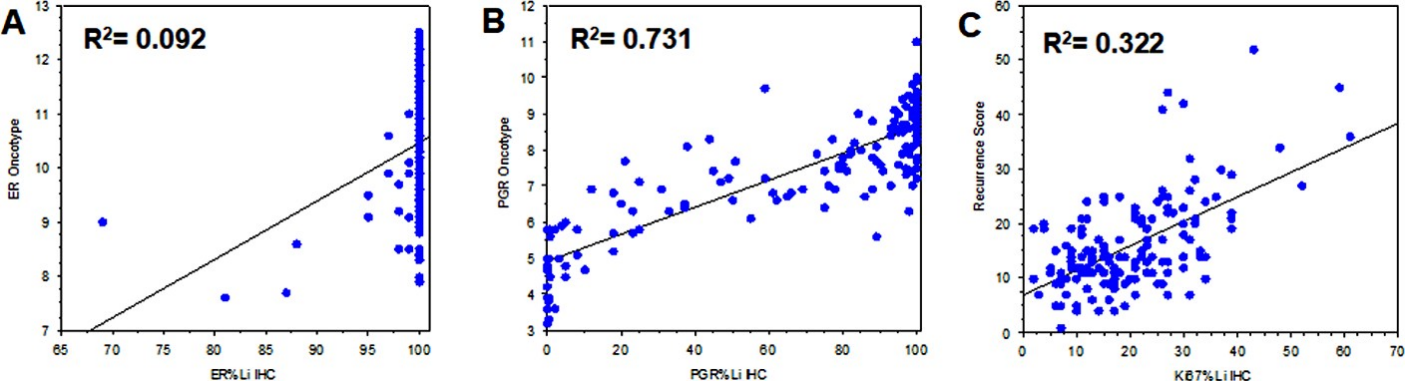
Bivariate Scatter Plots of ER, PGR, and Ki67 IHC versus Oncotype Dx. Bivariate Scatter Plots of (**A**) Estrogen Receptor IHC versus ER Oncotype Dx; (**B**) Progesterone Receptor IHC versus PGR Oncotype Dx; (**C**) Ki67 IHC versus Oncotype Dx Recurrence Score.

**Fig 2:**
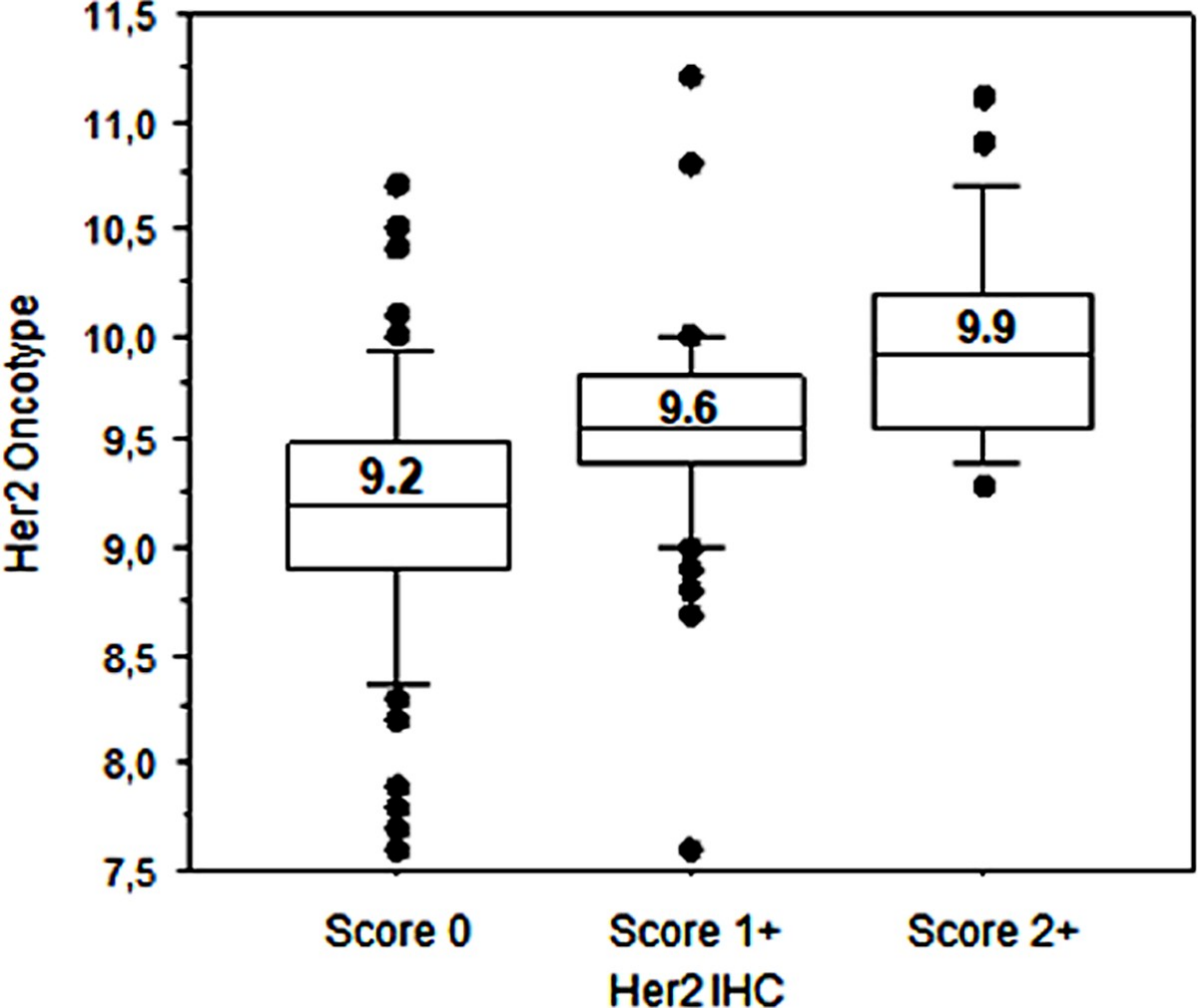
Box Plot analysis of Her2 IHC versus Her2 Oncotype Dx.

- All cases sent to Oncotype evaluation were confirmed ER-positive and Her2-negative by qRT-PCR assay. Considering ER%Li distribution, 139 (89.1%) cases showed all neoplastic cells positive for estrogen receptor expression with their corresponding ER-Dx values ranging from 7.9 to 12.5 units. These values encompassed the great majority of qRT-PCR positive range (6.5 - ≥12.5 units). No significant association was found between ER%Li and ER-Dx due to the high preponderance of 100% ER%Li (Fig 1A). Instead, a significant association was found between PGR%Li and PGR-Dx determination (Fig 1B). Of the 156 cases tested for Her2 IHC, 91 (58.3%) were Score 0, 46 (29.5%) were Score 1+, and 19 (12.2%) were Score 2+/ISH not amplified cases. Oncotype Her2 (Her2 Dx) determination classified 151 (96.8%) cases as Negative and 5 (3.2%) cases as Equivocal. Kruskal-Wallis test showed a relationship between Her2 IHC and Her2-Dx qRT-PCR values (S2 Table) clearly depicted using a Box Plot graphic display (Fig 2). In order to make the assessment of Her2 comparable between the two methodologies, we refined the Her2-IHC classification, considering Score 0/1+ or Score 2+/ISH not amplified as Her2 Negative, and Score 2+/ISH equivocal as Her2-IHC equivocal. The comparison between Her2-IHC thus reclassified (3 equivocal) and Her2-Dx (5 equivocal) shows 6 discordant cases as follows: 4 Her2-Dx Equivocal classified as Her2-IHC negative, 2 IHC Equivocal classified as Her2-Dx negative. This is in line with the observation reported by Dabbs et al. [24] on the different classification of equivocal cases between Her2 IHC/FISH and Oncotype Dx qRT-PCR. No significant association was found between reclassified Her2 IHC cases and RS (Mann-Whitney test Z = -1.007; p = 0.592). On the contrary a significant association was found when considering Her2 Dx classes (Mann-Whitney test Z = -2.048; p = 0.002).

A principal component analysis (PCA) conducted on ER, PGR and Her2 evaluated for either Oncotype or IHC tests confirms the strong inverse correlation between Oncotype RS and PGR levels already reported in the literature [25,26]. ER is faibly associated with RS in the IHC analysis but not in the Oncotype counterpart. Instead in both analyses was associated to Her2 as it appeared in the second Eigenvector (S1 Fig).

Overall, considering only these three markers, the presence of ER in our series simply associated with the "luminal" pattern, while the extent of PGR presence was mainly associated with risk-of-recurrence. PCA analysis confirmed Her2 IHC not associated to RS (S1 Fig). This observation, combined with the presence of a few equivocal Her2 IHC cases, their different classification compared to Her2 Dx, and especially the lack of any significant association between Negative/Equivocal cases and RS, led us to exclude Her2 in our IHC bio-profiles.

#### Ki67 and Bcl2 evaluation

- Information regarding the other two IHC markers (Ki67 and Bcl2) are not directly reported in the Oncotype schedule. For these markers RS is the only informative parameter from which to obtain any predictive value. We have therefore evaluated this aspect by extrapolating it directly from the results of the Oncotype Dx test on our dataset. Ki67%Li was moderately associated to RS (Fig 1C); Bcl2 IHC showed 125 (80.1%) High cases and 31 (19.9%) Low cases and was inversely related to RS, (S2 Table), with RS mean values of 14.2 for Bcl2-High cases and 21.7 for Bcl2-Low cases.

Multinomial ordinal logistic regression analysis confirmed Bcl2, PGR, and Ki67 as independent risk factors significantly correlated with traditional as well as TAILORx risk class classification (Table 2).

**Table 2:** Multiple multinomial logistic regression analysis of Oncotype risk class classification vs PGR, Ki67, and Bcl2.

Oncotype Traditional Risk-class High/Intermediate/Low Effect Likelihood Ratio Tests: Whole model test: ChiSquare = 91.749, p < 0.0001				
Source	Units	DF	L-R ChiSquare	P > ChiSq
Bcl2	High/Low	1	11.611	0.0007
PGR	%LI	1	34.766	< 0.0001
Ki67	%LI	1	39.298	< 0.0001
Oncotype TAILORx Risk-class High/Intermediate/Low Effect Likelihood Ratio Tests: Whole model test: ChiSquare = 78.479, p < 0.0001				
Source	Units	DF	L-R ChiSquare	P > ChiSq
Bcl2	High/Low	1	8.339	0.0039
PGR	%LI	1	29.266	< 0.0001
Ki67	%LI	1	31.159	< 0.0001

### Tailored predictive cut-off values for Ki67 and PGR IHC bio-markers

**Fig 3:**
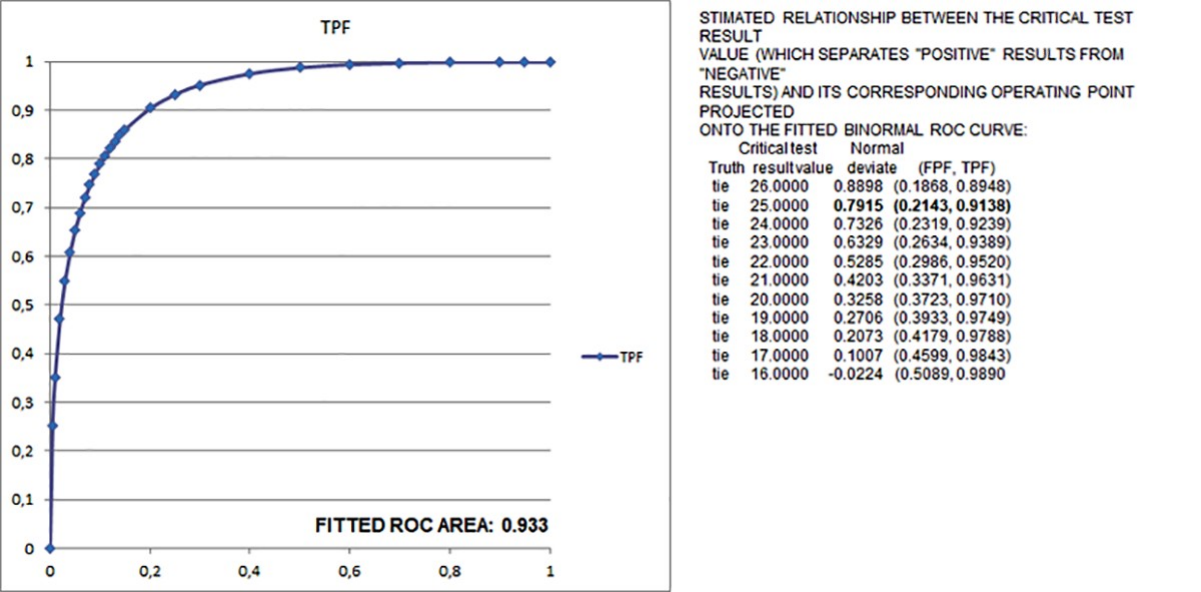
Binomial ROC curve for the identification of the most informative cut-off value for Ki67%Li. TPF (True Positive Fraction); FPF (False Positive Fraction).

Since PGR and to a less extent Ki67 emerged as informative parameters for this classification, we had to choose an appropriate cut-off value allowing us to correctly separate PGR negative versus positive cases and the high Ki67 cases from the low ones. Cut-off values derived from the literature [9,17,27] were all obtained in a prognostic context and do not display the needed predictive value. Consequently, we defined specific cut-off values for PGR and Ki67 according to the RS of the Oncotype Dx test. Comparing PGR-Dx risk classification with PGR%Li values, the cut-off that best reflects the distribution of risk classes was found to be 4%. Using this cut-off value, the comparison between the PGR IHC vs Dx classification showed a concordance in 147 (94.2%) cases (S2 Table). The distribution of the Oncotype Dx risk class for PGR IHC vs Dx showed 153 (98.1%) concordant cases for traditional classification, and 155 (99.4%) concordant cases for TAILORx classification (Table 3). In summary, the 4% PGR IHC cut-off value appears to be as informative as its Oncotype counterpart, especially for TAILORx classification. For Ki67%Li, the evaluation by ROC Plot Analysis of which could be the best cut-off value to distinguish high risk from intermediate/low risk cases (Fig 3) suggested 25% as the most informative value. Applying this value to our cases, all Oncotype Dx High risk patients had values of Ki67>25%Li (Ki67-High), while all those classified at Low/Intermediate risk had values of %Li≤25% (Ki67-Low), both for traditional and for TAILORx Oncotype (S3 Table).

**Table 3:** PGR IHC (4%Li cut-off value) and PGR Dx (5.5 cut-off value) RS risk class distribution.

**Low Risk** n° Cases	PGR Dx Neg	PGR Dx Pos	PGR IHC Neg	PGR IHC Pos	Δ
Traditional 106	5	101	6	100	1
TAILORx 40	0	40	0	40	0
**Intermediate Risk**	PGR Dx Neg	PGR Dx Pos	PGR IHC Neg	PGR IHC Pos	Δ
Traditional 42	11	31	9	33	2
TAILORx 102	14	88	13	89	1
**High Risk**	PGR Dx Neg	PGR Dx Pos	PGR IHC Neg	PGR IHC Pos	Δ
Traditional 8	5	3	5	3	0
TAILORx 14	7	7	7	7	0

Δ = n° of discordant cases

### Building and validating the predictive immunohistochemical profile

Taking in account these results, we tried to define which could be the main IHC profiles that can best predict the correct classification of early breast carcinoma according to the two main Oncotype Dx risk class categories: Oncotype Low/Intermediate risk of distant recurrence; Oncotype High risk of distant recurrence. For this purpose, we used an explorative analysis (correspondence analysis) carried out by combining the classification of bio-pathological parameters that we proved to be significantly associated with the Recurrence Score, (Bcl2 Low/High, PGR Neg/Pos, Ki67 Low/High), and the risk classes as defined by traditional and TAILORx Oncotype.

From this exploratory analysis combined Ki67-High/Bcl2-Low and Ki67-Low/Bcl2-High expression closely associated respectively with the high (H) and low (L) risk classes (S2 Fig). The expression of PGR presents a different behavior. PGR positive results are clearly associated to a Low/Intermediate risk, while a PGR negative result associated to Intermediate than High risk tumors (S2 Fig). Therefore, it seems that PGR is more indicative of the hormonal biological background of the tumor, while the other two parameters characterize more decisively the High and Low risk classes.

All this information suggested us to consider at least two different biological landscapes, a PGR-Neg and a PGR-Pos ones that we could define as luminal-A (Lum-A) and luminal-B (Lum-B), in which to put Bcl2 and Ki67 Low/High classification. As a result, for each luminal group we obtained 4 subgroups: Lum-A/B (no Bcl2/Ki67 alterations); Lum-A/B Bcl2 (Bcl2-Low); Lum-A/B Ki67 (Ki67-High); Lum-A/B Bcl2/Ki67 (Bcl2-Low/Ki67-High).

Both Lum-A and -B groups showed a growth of mean RS values from no alteration to the combined alteration of Bcl2/Ki67, with Lum-B subtypes having a higher mean RS value respective to their Lum-A counterpart, except for Bcl2 subtypes (Table 4).

**Table 4:** Oncotype Dx Recurrence Score (RS) and risk groups distribution among IHC subgroups.

Luminal IHC subgroup	N° cases	RS (mean ± S.D.)	Oncotype risk groups distribution Traditional TAILORx					
			L I H			L I H		
Lum-A	90	11.5 ± 4.7	82	8	0	36	54	0
Lum-A Bcl2	11	16.7 ± 6.6	5	6	0	1	10	0
Lum-A Ki67	20	18.2 ± 7.4	10	9	1	3	15	2
Lum-A Bcl2/Ki67	15	22.5 ± 6.4	3	10	2	0	10	5
Lum-B	11	18.9 ± 3.9	5	6	0	0	11	0
Lum-B Bcl2	2	17.5 ± 3.5	1	1	0	0	2	0
Lum-B Ki67	4	41.5 ± 9.5	0	1	3	0	0	4
Lum-B Bcl2/Ki67	3	39.0 + 7.9	0	1	2	0	0	3

Lum-A (luminal-A) = PGR < 4%, Lum-B (luminal-B) = PGR ≥ 4%; S.D. = Standard Deviation; L = Low risk; I = Intermediate risk; H = High risk

If we consider the distribution of Oncotype Dx Risk groups according to our immunohistochemical subgroups, we observed no High-risk cases present in the subgroups Lum-A/B, or in those with only Bcl2 alteration. Otherwise, considering Lum-A group, High-risk cases were predominantly located in the Lum-A Bcl2/Ki67 subgroup, while in Lum-B group High-risk cases were clustered in Lum-B Ki67 and Lum-B Bcl2/Ki67 subgroups (Table 4). Restriction of the analysis only to pN0 cases confirmed the above reported results, showing for Lum-A Bcl/Ki67 subgroup an even higher incidence of High-risk cases, especially using TAILORx classification (S4 Table).

By adapting the classification of IHC subgroups to that of Oncotype Dx, three main predictive classes are obtained: Low risk IHC class (Lum-A/B and Lum-A/B Bcl2); High risk IHC class (Lum-B Ki67 and Lum-B Bcl2/Ki67); Intermediate risk IHC class (Lum-A Ki67 and Lum-A Bcl2/Ki67). Overall accuracy is low (75% - 117/156 Oncotype Traditional; 44.2% - 69/156 TAILORx) due to the different classification of Low and Intermediate risk cases between IHC and Dx, but the predictive values are in line with our objective. Indeed, the predictive value of the Low risk IHC class for the presence of Oncotype High risk cases is 0% (0/114 cases) for both Traditional as well as TAILORx classification, whereas for the High risk IHC class it is 71.4% (5/7 cases) for Traditional and 100% (7/7 cases) for TAILORx (Table 4). The predictive value of the intermediate risk class IHC is low—8.6% (3/35 cases) Traditional, 20.0% (7/35 cases) TAILORx, thus confirming that the latter IHC subgroups include those cases that need to be sent to the Oncotype test for a correct classification of their risk.

Since we are currently unable to provide confirmation of data on a new set of patients, we cross-validated the results of the multinomial logistic regression by performing Jackknife resampling procedure that has confirmed the results. The yield of the three covariates by Jackknife cross-validation are reported in the S5 Table.

## Discussion

Many studies already exist in literature disputing about a simpler predictive value attributed to immunohistochemical bio-profile as opposed to the most expensive but validated Oncotype Dx assay. Of note, at least PGR and proliferation emerge as main players from these studies [13,25,26], in accordance to the predominant role in the RS algorithm presented by Paik et al. [2]. PGR IHC determination was already demonstrated equal to its Oncotype counterpart, especially when H-score method was used [28]. Moreover, when its expression is classified into Negative vs Positive cases an overall concordance between PGR IHC and Dx ranging from 85.8% to 91.3% was reported [28–32], and an inverse relation with RS is also convincingly demonstrated [28–31,33]. Our results are in line with these previously reported observations. Linear regression analysis showed a good relationship between PGR IHC and its molecular counterpart (R^2^ = 0.731). Principal component analysis on Oncotype or IHC ER, PGR, Her2 and RS confirmed the strong inverse relation between RS and PGR [25,26], and multinomial logistic regression analysis demonstrated an independent association to RS predictive risk-of-recurrence value for PGR IHC. Estrogen Receptor seems to play a minor role in this situation also if a relationship between ER IHC and its Oncotype counterpart was reported [28]. In our cases, the comparison between IHC and Oncotype ER and PGR values showed a relationship for PGR, but not for ER. Moreover, principal component analysis showing the absence of correlation between ER IHC and RS, suggests that the predictive role of ER IHC is irrelevant here, contributing this latter only to define cases as “luminal”.

Ki67 immunohistochemical determination was already shown to directly correlate with RS, despite the difficulty of comparing the reported results due to different scoring methods and cut-off values [11–13,34]. In our series we found a moderate relationship between Ki67 IHC and RS (R^2^ = 0.322). Despite this, we demonstrated a strong and independent relationship between Ki67 and RS (Table 2), both for traditional and TAILORx risk class classification.

As regards the determination of Her2, the results obtained are clearly contradictory. In fact, although there is a significant association between Her2 IHC and the respective qRT-PCR values (Fig 2; S2 Table), the multivariate PCA analysis does not show any significant association with RS (S1 Fig). The same lack of association is detectable by considering the classification of Her2 in Negative vs. Equivocal, unlike its counterpart Her2 Dx, which is directly related to RS (Mann-Whitney test Z = -2.048; p = 0.002). That there may be a discrepancy between Her2 IHC and Dx is already reported by Dabbs et al. [24], particularly in the classification of Equivocal cases. It should also be noted that the poor representativeness of the latter class in our series (3 cases of IHC, 5 cases of Oncotype), may have accentuated this discrepancy. In the light of these considerations we have excluded this parameter from the evaluation of the IHC bio-profile as predictor of the risk classes Oncotype Dx.

In clinical practice, in order to predict the need of chemotherapy in luminal cases, ER, PGR, and Ki67 are not separately considered, but combined together in the attempt to distinguish luminal-A from -B bio-profiles. The most convincing evidence that combination, rather than single consideration, may be the right approach was the “IHC4” score proposed by Cuzick et al, demonstrating on TransATAC trial dataset the ability to predict outcome better than Oncotype Dx [10]. According to ASCO/CAP and St.Gallen recommendations, luminal-B bio-profile encompasses all cases Her2-negative with a low ER and/or PGR expression. What we have to intend with the term “low” is already debatable, because at least three different cut-off values (≤1%-20%-10%) were proposed [5–8]. Ki67 cut-off values suffer the same drawback, ranging from 14% to 20–30% depending on what recommendation we take in account [9,14–17]. Whatever the selected value is, papers dealing with IHC vs Oncotype Dx risk-of-recurrence predictive value all considered an “a priori” (prognostic) cut-off value, instead of defining one clipped on Recurrence Score results.

To integrate PGR and Ki67 into a simple predictive bio-profile we have defined specific cut-off values, tailored on Oncotype RS. For PGR IHC it means that we need a value that mirrors the same risk class distribution of its Oncotype counterpart. We found 4% as the cut-off value corresponding to this necessity. Using this value, concordance between PGR IHC and Dx Negative vs Positive cases was 94.2%, a very good result regarding to the literature [28–32]. Most important, comparing PGR IHC or Dx classification of cases using Oncotype Risk class distribution, 153/156 (98.1%) cases showed the same attribution using traditional risk class, and only 1 case was discordant following the recently proposed TAILORx cut-off values (Table 3). In brief, PGR IHC 4% cut-off value seems to be as informative as its Oncotype counterpart.

For Ki67 evaluation, we confirmed its independent relationship with Oncotype RS, as demonstrated by principal component and logistic regression analysis, and as for PGR, to integrate its predictive value in the IHC bio-profile we select a proper cut-off value. ROC curve analysis showed 25%Li as most appropriate to divide High risk cases from the Intermediate/Low ones (Fig 3).

Very few papers reported Bcl2 as a possible partner of these bio-profiles, despite it was clearly demonstrated as a significant prognostic marker in breast cancer [18–21], and is part of the hormonal-related Oncotype Dx signature. Bcl2 is added to our analysis taking part of our IHC bio-profile until 1995. Bcl2-IHC determination showed a significant inverse relation with RS (S2 Table), and multinomial logistic regression analysis confirmed its independent association with Oncotype traditional and TAILORx risk class classification.

At the best of our knowledge this is the first time that Bcl2 is reported as an independent significant predictor of Oncotype risk-of-recurrence score.

Based on these findings, we have built specific IHC bio-profiles tailored on Oncotype Risk-of-recurrence classification. Correspondence analysis suggested us a “landscape” role for PGR-IHC, identifying two large groups, PGR-Positive (Lum-A) and PGR-Negative (Lum-B), on which to fall the other indicators (Bcl2, Ki67). Therefore, each group was split into four subgroups: no alteration (Lum-A/B); Low Bcl2 (Lum-A/B Bcl); High Ki67 (Lum-A/B Ki67); combined Bcl2 Low and High Ki67 (Lum-A/B Bcl/Ki67). Considering the distribution of High-risk cases defined by Oncotype traditional as well as TAILORx RS cut-off values, only luminal Ki67-High or combined Bcl2-Low/Ki67-High subgroups shared their presence, with the last ones having the higher incidence of High-risk cases, especially when TAILORx classification was applied and irrespective of the pN status (Table 4, S4 Table).

It is necessary to note that there are some limitations to our study. The number of cases considered is rather small, and may be that the irrelevance of ER and Her2 IHC here found is related to this drawback. Moreover, the suggestions we have reported are conditioned by an observer-dependent evaluation of the IHC parameters, especially for ER and PGR (no H-score), also if for PGR IHC this seems not so relevant.

Finally, and most importantly, we know that for the validation of this IHC predictor it is necessary to confirm these results on a further dataset of patients. The collection of a sufficient number of cases is ongoing but it will require quite a long time. However, Jackknife resampling procedure cross-validation of our results may strongly suggest in our opinion that IHC profiles built using the proposed methodology may be a useful predictor of Oncotype Dx categories.

## Conclusions

In conclusion, we have shown that for the best selection of cases to be submitted to the Oncotype Dx test it is advantageous to add Bcl2 to the IHC bio-profile and to select on its own dataset PGR and Ki67 cut-off values tailored on Oncotype Dx results rather than to apply “prognostic” cut-off values. If these results will be confirmed in the new patient dataset being collected, the addition of Bcl2 to IHC bio-profiles will strongly suggest to submit only those PGR-positive Ki67 or Bcl2/Ki67 altered cases to Oncotype Dx test, and directly indicate the need of chemotherapy for those PGR-Negative Ki67 or Bcl2/Ki67 altered cases, showing this immunohistochemical bio-profiles as a useful pre-selection tool for Oncotype Dx referral at the time of pathological diagnosis.

## Supporting information

S1 Fig PCA/FA analyses including RS + IHC markers (left) and RS + Oncotype markers (right).
(DOCX)

S2 Fig Multiple Correspondence analysis for main IHC predictive bio-profiles. (DOCX)

S1 Table Immunohistochemical tests. (DOCX)

S2 Table Confirmatory statistical analyses for immunohistochemical Her2, Bcl2, and PGR. (DOCX)

S3 Table Dataset. (XLS)

S4 Table Oncotype Dx Recurrence Score (RS) and Risk groups distribution for pN0 patients. (DOCX)

S5 Table Jackknife resampling procedure results. (DOCX)
