# Supplementary material for: A simple immunohistochemical bio-profile incorporating Bcl2 curbs those cases of invasive breast carcinoma for which an Oncotype Dx characterization is needed
Source: PLoS One. 2019 Jun 3;14(6):e0217937. doi: 10.1371/journal.pone.0217937 (PMC6546245; doi:10.1371/journal.pone.0217937)
Supplement: S1 Fig — PCA/FA analyses including RS + IHC markers (left) and RS + Oncotype markers (right). (DOCX) [file pone.0217937.s001.docx]

S3 Fig. PCA/FA analyses including RS + IHC markers (left) and RS + Oncotype markers (right).


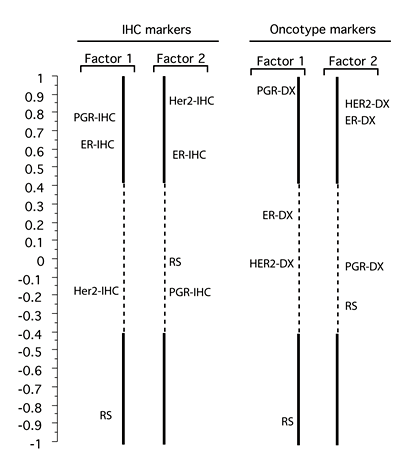


For the RS + IHC markers analysis the first two eingenvalues were 21.75 and 1.09, including 43.7 and 29.1 of total variance. For the RS + Oncotype markers analysis, the first two eingenvalues were 1.69 and 1.25, resuming 42.4 and 31.2 of total variance, respectively. Variables are visually associated to the axis of factors 1 and 2 and are aligned with their factor loading value, whose scale is shown on the left. The variables which fall in the interval of factor loadings between 0.4 and - 0.4. (dotted lines) are not useful for factor interpretation.
