# Supplementary material for: A simple immunohistochemical bio-profile incorporating Bcl2 curbs those cases of invasive breast carcinoma for which an Oncotype Dx characterization is needed
Source: PLoS One. 2019 Jun 3;14(6):e0217937. doi: 10.1371/journal.pone.0217937 (PMC6546245; doi:10.1371/journal.pone.0217937)
Supplement: S2 Fig — (DOCX) [file pone.0217937.s002.docx]

S5 Fig. Multiple Correspondence analysis for main IHC predictive bio-profiles.


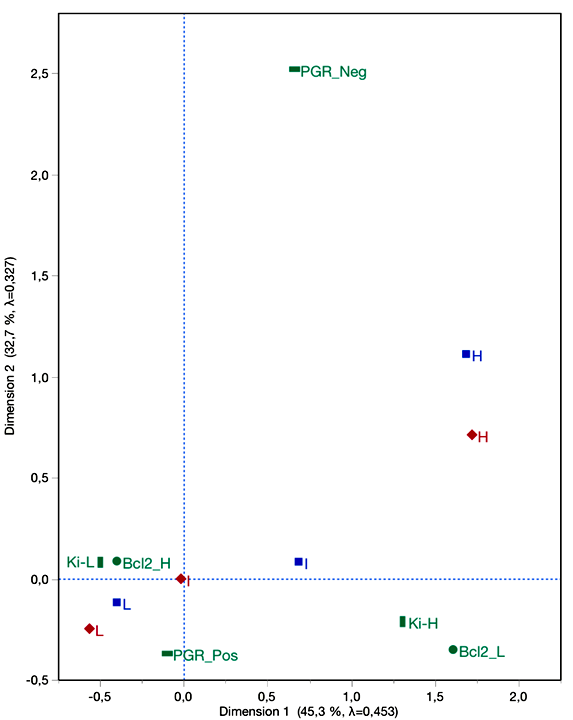


The first two axes of multiple correspondence analysis using immunohistochemical markers Ki-67 (low/high), Bcl2 (low/high), and PGR (neg/pos) are plotted in the figure. The two axes account for an inertia of 0,78, with the first axis accounting for 45,3% and the second for 32.75% of the total inertia. All points display satisfactory quality, varying between 0.66 and 0.99. Therefore, all points are quite well represented in the above two dimensional map. Low (L), intermediate (I) and high (H) Oncotype DX risk classes (blu squares) and Taylor risk classes (red diamonds) were added to the map as supplementary variables.
