# Supplementary material for: A simple immunohistochemical bio-profile incorporating Bcl2 curbs those cases of invasive breast carcinoma for which an Oncotype Dx characterization is needed
Source: PLoS One. 2019 Jun 3;14(6):e0217937. doi: 10.1371/journal.pone.0217937 (PMC6546245; doi:10.1371/journal.pone.0217937)
Supplement: S1 Table — (DOCX) [file pone.0217937.s003.docx]

| S1 Table: Immunohistochemical tests | | | | |
| --- | --- | --- | --- | --- |
| **Test** | **Antibody (clone and Source)** | **Method**  **AR Ab Incubation Detection Kit** | | |
| ER | SP1 Ventana | UltraCC1 x 32 Min - 95°C | 24 Min – 36°C | OptiView DAB |
| PGR | 1E2 Ventana | UltraCC1 x 32 Min - 95°C | 12 Min – 36°C | OptiView DAB |
| Ki-67 | 30-9 Ventana | UltraCC1 x 32 Min - 95°C | 8 Min – RT | OptiView DAB |
| HER2 | 4B5 Ventana | UltraCC1 x 36 Min - 95°C | 16 Min – 36°C | UltraView DAB |
| Bcl2 | SP66 Ventana | UltraCC1 x 32 Min - 95°C | 16 Min – 36°C | OptiView DAB |

AR = Antigen retrieval; UltraCC1 = Tris-HCl Buffer pH 8.2; RT = Room Temperature
