# Supplementary material for: A simple immunohistochemical bio-profile incorporating Bcl2 curbs those cases of invasive breast carcinoma for which an Oncotype Dx characterization is needed
Source: PLoS One. 2019 Jun 3;14(6):e0217937. doi: 10.1371/journal.pone.0217937 (PMC6546245; doi:10.1371/journal.pone.0217937)
Supplement: S2 Table — (DOCX) [file pone.0217937.s004.docx]

S2 Table: Confirmatory statistical analyses for IHC Her2, Bcl2, and PGR

| 1. **Association Between Her2 IHC Score and Her2 Dx qRT-PCR values** | | | | |
| --- | --- | --- | --- | --- |
| **Kruskal-Wallys test: H = 35.288; p < 0.0001** | | | | |
|  | Count | Sum Ranks | Mean Rank |  |
| Her2 IHC Score 0 | 91 | 5616.0 | 61.714 |  |
| Her2-IHC Score 1+ | 46 | 4316.5 | 93.837 |  |
| Her2-IHC Score 2+ | 19 | 2313.5 | 121.763 |  |

| 1. **Association Between Bcl2 IHC Low/High and Oncotype RS values** | | | | |
| --- | --- | --- | --- | --- |
| **Mann-Whitney test: Z = -4.612; p < 0.0001** | | | | |
|  | Count | Sum Ranks | Mean Rank |  |
| Bcl2 IHC Low | 31 | 8774.0 | 170.192 |  |
| Bcl2 IHC High | 125 | 3472.0 | 112.0 |  |

| 1. **Association between PGR IHC Neg/Pos and PGR Dx Neg/Pos cases** | | | |
| --- | --- | --- | --- |
| **Fisher’s exact test: chi-square = 87.188; p < 0.0001** | | | |
|  | PGR Dx Neg | PGR Dx Pos |  |
| PGR IHC Neg | 16 | 4 |  |
| PGR IHC Pos | 5 | 131 |  |
