# Supplementary material for: A simple immunohistochemical bio-profile incorporating Bcl2 curbs those cases of invasive breast carcinoma for which an Oncotype Dx characterization is needed
Source: PLoS One. 2019 Jun 3;14(6):e0217937. doi: 10.1371/journal.pone.0217937 (PMC6546245; doi:10.1371/journal.pone.0217937)
Supplement: S4 Table — (DOCX) [file pone.0217937.s006.docx]

| S6 Table: Oncotype Dx Recurrence Score (RS) and Risk groups distribution for pN0 patients | | | | | | | | |
| --- | --- | --- | --- | --- | --- | --- | --- | --- |
| **Luminal IHC subgroup** | **N° cases** | **RS (mean + S.D.)** | **Oncotype risk groups distribution**  **Traditional TAILORx** | | | | | |
|  |  |  | **L I H** | | | **L I H** | | |
| Lum-A | 67 | 11.6 + 4.5 | 62 | 5 | 0 | 26 | 41 | 0 |
| Lum-A Bcl2 | 5 | 14.0 + 7.5 | 3 | 2 | 0 | 1 | 4 | 0 |
| Lum-A Ki67 | 13 | 17.5 + 6.4 | 7 | 6 | 0 | 2 | 10 | 1 |
| Lum-A Bcl2/Ki67 | 9 | 24.1 + 5.9 | 1 | 7 | 1 | 0 | 5 | 4 |
| Lum-B | 9 | 18.4 + 4.2 | 5 | 4 | 0 | 0 | 9 | 0 |
| Lum-B Bcl2 | 2 | 17.5 + 3.5 | 1 | 1 | 0 | 0 | 2 | 0 |
| Lum-B Ki67 | 1 | 41.0 | 0 | 0 | 1 | 0 | 0 | 1 |
| Lum-B Bcl2/Ki67 | 2 | 43.5 + 2.1 | 0 | 0 | 2 | 0 | 0 | 2 |

Lum-A = PGR < 4%, Lum-B = PGR > 4%; S.D. = Standard Deviation; L = Low risk; I = Intermediate risk;

H = High risk
