# Supplementary material for: A simple immunohistochemical bio-profile incorporating Bcl2 curbs those cases of invasive breast carcinoma for which an Oncotype Dx characterization is needed
Source: PLoS One. 2019 Jun 3;14(6):e0217937. doi: 10.1371/journal.pone.0217937 (PMC6546245; doi:10.1371/journal.pone.0217937)
Supplement: S5 Table — (DOCX) [file pone.0217937.s007.docx]

S7 Table: Jackknife resampling procedure results

Stata

Ordered logistic regression Number of obs = 156

Replications = 156

F( 3, 155) = 13.49

Prob > F = 0.0000

Log likelihood = -73.96099 Pseudo R2 = 0.3828

------------------------------------------------------------------------------

| Jackknife

RiskOncoDX | Coef. Std. Err. t P>|t| [95% Conf. Interval]

-------------+----------------------------------------------------------------

bcl_2_2 | 1.648861 .568776 2.90 0.004 .5253085 2.772414

pgrli | -.0327695 .0066241 -4.95 0.000 -.0458546 -.0196844

ki67li | .1322205 .0266586 4.96 0.000 .0795594 .1848815

-------------+----------------------------------------------------------------

/cut1 | 1.825251 .6217717 .597011 3.053491

/cut2 | 5.658917 .9302664 3.82128 7.496553

------------------------------------------------------------------------------

Ordered logistic regression Number of obs = 156

Replications = 156

F( 3, 155) = 17.71

Prob > F = 0.0000

Log likelihood = -92.288584 Pseudo R2 = 0.2983

------------------------------------------------------------------------------

| Jackknife

RiskTailorA | Coef. Std. Err. t P>|t| [95% Conf. Interval]

-------------+----------------------------------------------------------------

bcl_2_2 | 1.532116 .5472115 2.80 0.006 .4511612 2.61307

pgrli | -.0296894 .0063736 -4.66 0.000 -.0422797 -.0170991

ki67li | .1099218 .0228035 4.82 0.000 .064876 .1549676

-------------+----------------------------------------------------------------

/cut1 | -1.382053 .5877001 -2.542988 -.2211178

/cut2 | 4.133704 .6489682 2.851741 5.415667

------------------------------------------------------------------------------

**. jackknife: ologit risktailorA pgrli**

(running ologit on estimation sample)

Jackknife replications (156)

----+--- 1 ---+--- 2 ---+--- 3 ---+--- 4 ---+--- 5

.................................................. 50

.................................................. 100

.................................................. 150

......

Ordered logistic regression Number of obs = 156

Replications = 156

F( 1, 155) = 21.43

Prob > F = 0.0000

Log likelihood = -115.83165 Pseudo R2 = 0.1193

------------------------------------------------------------------------------

| Jackknife

risktailorA | Coef. Std. Err. t P>|t| [95% Conf. Interval]

-------------+----------------------------------------------------------------

pgrli | -.0284934 .0061546 -4.63 0.000 -.040651 -.0163358

-------------+----------------------------------------------------------------

/cut1 | -3.244957 .5304678 -4.292836 -2.197078

/cut2 | .9369049 .3328271 .2794426 1.594367

------------------------------------------------------------------------------

**. jackknife: ologit risktailorA ki67li**

(running ologit on estimation sample)

Jackknife replications (156)

----+--- 1 ---+--- 2 ---+--- 3 ---+--- 4 ---+--- 5

.................................................. 50

.................................................. 100

.................................................. 150

......

Ordered logistic regression Number of obs = 156

Replications = 156

F( 1, 155) = 35.46

Prob > F = 0.0000

Log likelihood = -112.70324 Pseudo R2 = 0.1431

------------------------------------------------------------------------------

| Jackknife

risktailorA | Coef. Std. Err. t P>|t| [95% Conf. Interval]

-------------+----------------------------------------------------------------

ki67li | .1079395 .0181253 5.96 0.000 .0721351 .1437438

-------------+----------------------------------------------------------------

/cut1 | .7058455 .3123642 .0888052 1.322886

/cut2 | 4.919141 .5056028 3.92038 5.917902

------------------------------------------------------------------------------

**. xi: jackknife: ologit risktailorA i.bcl_2**

i.bcl_2 _Ibcl_2_1-2 (_Ibcl_2_1 for bcl_2==High omitted)

(running ologit on estimation sample)

Jackknife replications (156)

----+--- 1 ---+--- 2 ---+--- 3 ---+--- 4 ---+--- 5

.................................................. 50

.................................................. 100

.................................................. 150

......

Ordered logistic regression Number of obs = 156

Replications = 156

F( 1, 155) = 17.06

Prob > F = 0.0001

Log likelihood = -121.36429 Pseudo R2 = 0.0773

------------------------------------------------------------------------------

| Jackknife

risktailorA | Coef. Std. Err. t P>|t| [95% Conf. Interval]

-------------+----------------------------------------------------------------

_Ibcl_2_2 | 2.114048 .5118151 4.13 0.000 1.103015 3.125081

-------------+----------------------------------------------------------------

/cut1 | -.8067264 .1947045 -1.191343 -.4221096

/cut2 | 3.095833 .4346643 2.237203 3.954463

------------------------------------------------------------------------------

**SIMPLE LOGISTIC MODEL FOR RISK (Traditional Oncotype classification)**

**xi: jackknife _b: logit riskA i.bcl_2 pgrli ki67li**

i.bcl_2 _Ibcl_2_1-2 (_Ibcl_2_1 for bcl_2==High omitted)

(running logit on estimation sample)

Jackknife replications (156)

----+--- 1 ---+--- 2 ---+--- 3 ---+--- 4 ---+--- 5

.................................................. 50

.................................................. 100

.................................................. 150

......

Logistic regression Number of obs = 156

Replications = 156

F( 3, 155) = 9.27

Prob > F = 0.0000

Log likelihood = -59.943574 Pseudo R2 = 0.3874

------------------------------------------------------------------------------

| Jackknife

riskA | Coef. Std. Err. t P>|t| [95% Conf. Interval]

-------------+----------------------------------------------------------------

_Ibcl_2_2 | 1.972222 .6579415 3.00 0.003 .6725326 3.271911

pgrli | -.0299052 .0068441 -4.37 0.000 -.0434249 -.0163854

ki67li | .1158149 .0291335 3.98 0.000 .0582649 .1733649

_cons | -1.74631 .6670949 -2.62 0.010 -3.064081 -.4285397

------------------------------------------------------------------------------

**SIMPLE LOGISTIC MODEL FOR RISK (TAILORx Oncotype classification)**

**xi: jackknife _b: logit risktailorA i.bcl_2 pgrli ki67li**

i.bcl_2 _Ibcl_2_1-2 (_Ibcl_2_1 for bcl_2==High omitted)

(running logit on estimation sample)

Jackknife replications (156)

----+--- 1 ---+--- 2 ---+--- 3 ---+--- 4 ---+--- 5

...n.............................................. 50

.................................................. 100

.................................................. 150

......

Logistic regression Number of obs = 156

Replications = 155

F( 3, 154) = 20.57

Prob > F = 0.0000

Log likelihood = -66.976016 Pseudo R2 = 0.2458

------------------------------------------------------------------------------

| Jackknife

risktailorA | Coef. Std. Err. t P>|t| [95% Conf. Interval]

-------------+----------------------------------------------------------------

_Ibcl_2_2 | 2.110051 .3715155 5.68 0.000 1.376127 2.843976

pgrli | -.0353994 .0115318 -3.07 0.003 -.0581805 -.0126184

ki67li | .0527008 .0233802 2.25 0.026 .0065135 .0988881

_cons | 2.743291 1.098573 2.50 0.014 .5730738 4.913509

------------------------------------------------------------------------------

Note: one or more parameters could not be estimated in 1 jackknife replicate;

standard error estimates include only complete replications.

**LINEAR REGRESSION MODEL FOR RISK (Traditional Oncotype classification)**

**. xi: jackknife _b: regress riskA i.bcl_2 pgrli ki67li**

i.bcl_2 _Ibcl_2_1-2 (_Ibcl_2_1 for bcl_2==High omitted)

(running regress on estimation sample)

Jackknife replications (156)

----+--- 1 ---+--- 2 ---+--- 3 ---+--- 4 ---+--- 5

.................................................. 50

.................................................. 100

.................................................. 150

......

Linear regression Number of obs = 156

Replications = 156

F( 3, 155) = 38.32

Prob > F = 0.0000

R-squared = 0.4901

Adj R-squared = 0.4801

Root MSE = 0.4194

------------------------------------------------------------------------------

| Jackknife

riskA | Coef. Std. Err. t P>|t| [95% Conf. Interval]

-------------+----------------------------------------------------------------

_Ibcl_2_2 | .3231431 .1098977 2.94 0.004 .1060525 .5402336

pgrli | -.0056076 .0011414 -4.91 0.000 -.0078624 -.0033529

ki67li | .0235159 .0042204 5.57 0.000 .0151789 .0318528

_cons | .2300061 .1272087 1.81 0.073 -.0212803 .4812926

------------------------------------------------------------------------------.

**LINEAR REGRESSION MODEL FOR RISK (TAILORx Oncotype classification)**

**xi: jackknife _b: regress risktailorA i.bcl_2 pgrli ki67li**

i.bcl_2 _Ibcl_2_1-2 (_Ibcl_2_1 for bcl_2==High omitted)

(running regress on estimation sample)

Jackknife replications (156)

----+--- 1 ---+--- 2 ---+--- 3 ---+--- 4 ---+--- 5

.................................................. 50

.................................................. 100

.................................................. 150

......

Linear regression Number of obs = 156

Replications = 156

F( 3, 155) = 33.36

Prob > F = 0.0000

R-squared = 0.3900

Adj R-squared = 0.3780

Root MSE = 0.4464

------------------------------------------------------------------------------

| Jackknife

risktailorA | Coef. Std. Err. t P>|t| [95% Conf. Interval]

-------------+----------------------------------------------------------------

_Ibcl_2_2 | .2633056 .0883427 2.98 0.003 .0887947 .4378166

pgrli | -.005191 .0008206 -6.33 0.000 -.0068119 -.0035701

ki67li | .0199091 .0033575 5.93 0.000 .0132767 .0265416

_cons | .7452457 .0781789 9.53 0.000 .5908122 .8996793

------------------------------------------------------------------------------

**MULTINOMIAL LOGISTIC MODEL FOR RISK (Traditional Oncotype classification)**

**. xi: jackknife _b: mlogit riskA i.bcl_2 pgrli ki67li**

i.bcl_2 _Ibcl_2_1-2 (_Ibcl_2_1 for bcl_2==High omitted)

(running mlogit on estimation sample)

Jackknife replications (156)

----+--- 1 ---+--- 2 ---+--- 3 ---+--- 4 ---+--- 5

.................................................. 50

.................................................. 100

.................................................. 150

......

Multinomial logistic regression Number of obs = 156

Replications = 156

F( 6, 155) = 4.76

Prob > F = 0.0002

Log likelihood = -71.33493 Pseudo R2 = 0.4047

------------------------------------------------------------------------------

| Jackknife

riskA | Coef. Std. Err. t P>|t| [95% Conf. Interval]

-------------+----------------------------------------------------------------

1 |

_Ibcl_2_2 | 1.963309 .6503034 3.02 0.003 .6787082 3.24791

pgrli | -.0284149 .0070266 -4.04 0.000 -.0422951 -.0145346

ki67li | .1081318 .0314488 3.44 0.001 .0460082 .1702554

_cons | -1.711401 .6834338 -2.50 0.013 -3.061448 -.3613549

-------------+----------------------------------------------------------------

2 |

_Ibcl_2_2 | 2.038071 1.67076 1.22 0.224 -1.262327 5.338469

pgrli | -.0787254 .0388235 -2.03 0.044 -.1554169 -.0020339

ki67li | .2983315 .1018925 2.93 0.004 .0970544 .4996086

_cons | -7.833499 2.626658 -2.98 0.003 -13.02217 -2.644832

------------------------------------------------------------------------------

(riskA==0 is the base outcome)

**MULTINOMIAL LOGISTIC MODEL FOR RISK (TAILORx Oncotype classification)**

**. xi: jackknife: mlogit risktailorA i.bcl_2 pgrli ki67li, baseoutcome(0)**

i.bcl_2 _Ibcl_2_1-2 (_Ibcl_2_1 for bcl_2==High omitted)

(running mlogit on estimation sample)

Jackknife replications (156)

----+--- 1 ---+--- 2 ---+--- 3 ---+--- 4 ---+--- 5

.................................................. 50

.................................................. 100

.................................................. 150

......

Multinomial logistic regression Number of obs = 156

Replications = 156

F( 6, 155) = 5.03

Prob > F = 0.0001

Log likelihood = -85.799639 Pseudo R2 = 0.3477

------------------------------------------------------------------------------

| Jackknife

risktailorA | Coef. Std. Err. t P>|t| [95% Conf. Interval]

-------------+----------------------------------------------------------------

1 |

_Ibcl_2_2 | 2.067425 20.14685 0.10 0.918 -37.73041 41.86526

pgrli | -.0348741 .0116569 -2.99 0.003 -.0579009 -.0118472

ki67li | .0464002 .0261115 1.78 0.078 -.0051801 .0979805

_cons | 2.781061 1.113031 2.50 0.014 .5823942 4.979728

-------------+----------------------------------------------------------------

2 |

_Ibcl_2_2 | 3.233786 20.15793 0.16 0.873 -36.58592 43.0535

pgrli | -.0638246 .0206899 -3.08 0.002 -.104695 -.0229541

ki67li | .2711469 .0624244 4.34 0.000 .1478346 .3944593

_cons | -4.540663 2.042401 -2.22 0.028 -8.575196 -.5061297

----------------------------------------------------------------------------------------------------------------------------------

(risktailorA==0 is the base outcome)
